# Single-molecule protein identification by sub-nanopore sensors

**DOI:** 10.1371/journal.pcbi.1005356

**Published:** 2017-05-09

**Authors:** Mikhail Kolmogorov, Eamonn Kennedy, Zhuxin Dong, Gregory Timp, Pavel A. Pevzner

**Affiliations:** 1 Department of Computer Science and Engineering, University of California San Diego, La Jolla, California, United States of America; 2 Electrical Engineering and Biological Science, University of Notre Dame, Notre Dame, Indiana, United States of America; University of North Carolina at Chapel Hill, UNITED STATES

## Abstract

Recent advances in top-down mass spectrometry enabled identification of intact proteins, but this technology still faces challenges. For example, top-down mass spectrometry suffers from a lack of sensitivity since the ion counts for a single fragmentation event are often low. In contrast, nanopore technology is exquisitely sensitive to single intact molecules, but it has only been successfully applied to DNA sequencing, so far. Here, we explore the potential of sub-nanopores for single-molecule protein identification (SMPI) and describe an algorithm for identification of the electrical current blockade signal (nanospectrum) resulting from the translocation of a denaturated, linearly charged protein through a sub-nanopore. The analysis of identification p-values suggests that the current technology is already sufficient for matching nanospectra against small protein databases, e.g., protein identification in bacterial proteomes.

## Author summary

Protein identification is the key step in many proteomics studies. Currently, the most popular technique for intact protein analysis is top-down mass spectrometry which recently enabled high-throughput identification of many proteins and their proteoforms. However, this approach requires large amounts of materials and is currently limited to short proteins, typically less than 30 kDa. On the other hand, nanopore sensors promise single molecule sensitivity in protein analysis, but an approach for the identification of a single protein from its blockade current (nanospectrum) has remained elusive, since the signal from the sensors relates to the amino acid sequence of the protein in a poorly understood way. In this work we describe the first algorithm for protein identification based on nanospectra associated with translocation of proteins through pores with sub-nanometer diameters. While identification accuracy currently does not allow reliable processing of complex protein samples yet, we believe, that the rapidly improving experimental protocols along with the new computational algorithms will transform into a viable protein identification approach in the near future.

## Introduction

When Church et al. [1] proposed to use nanopores for sequencing biopolymers, they had envisioned *both* DNA and proteins sequencing. However, the progress in protein sequencing turned out to be much slower since it is more difficult to force proteins through a pore systematically and measure the resulting signal [2]. These difficulties underlay the experimental and computational challenges of *Single Molecule Protein Identification* (*SMPI*).

Nanopores promise single molecule sensitivity in the analysis of proteins, but an approach for the identification of a single protein from its nanospectrum has remained elusive. The most common approach to nanopore sequencing relies on the detection of the ionic –current blockade signal (nanospectrum) that develops when a molecule is driven through the pore by an electric field. Preliminary work [3, 4] was limited to analyzing protein conformations in pure solutions rather than identifying proteins in a mixture. Subsequent steps demonstrated that nanopores can detect protein phosphorylations [5] as well as conformations and protein-ligand interactions [6]. Recent studies on combining nanopores with aptamers have shown limited success for protein analysis [7]. Proposals for electrolytic cell with tandem nanopores and for single molecule protein sequencing have been made, but not yet implemented [8–11].

Recently, the sequence of amino acids in a denatured protein were read with limited resolution using a sub-nanometer-diameter pore, sputtered through a thin silicon nitride membrane [12]. Protein translocations through the pore modulated the measured ionic current, which was correlated with the volumes of amino adids in the proteins. However, the correlation was imperfect, making it difficult to solve the problem of reconstructing a protein from its nanospectrum with high fidelity.

Developing computational and experimental methods for analyzing nanospectra derived from a electrical signals that produced when a protein translocates through a sub-nanopore could enable a real-time sensitive approach to SMPI that may have advantages over top-down mass spectrometry for protein identification. Despite difficulty and expense (requiring especially powerful magnets) to implement it, top-down mass spectrometry has been used in a few labs around the world to identify intact proteins and their proteoforms. However, it is about 100-fold less sensitive than bottom-up mass spectrometry, which can be used to detect attomoles of material [13]. In stark contrast, a sub-nanopore has been used to discriminate residue substitutions in a *single* molecule with low fidelity [12].

Similar to mass-spectrometry, where *de novo* protein sequencing (based on top-down spectra) remains error-prone [14, 15], the challenge of *de novo* deconvoluting nanospectra into amino acids sequences of proteins is currently unsolved. However, protein identification based on top-down spectra (i.e., matching a spectrum against all proteins in a protein database) is a well-studied topic. For example, top-down protein identification tools ProsightPC [16] and MS-Align+ [17] reliably identify proteins, report p-values of resulting Protein-Spectrum Matches (PrSMs), and even contribute to improving gene annotations by discovering previously unknown proteins [18].

In this paper, we describe the first algorithm for protein identification based on nanospectra derived from current blockades associated with denaturated, charge linearized translocation of protein through pores with sub-nanometer diameters. Our Nano-Align algorithm matches nanospectra against a protein database, identifies Protein-Nanospectrum Matches (PrNMs), and reports their p-values. Our analysis revealed that the typical p-values of identified PrNMs vary from 10^−4^ to 10^−6^, which is already sufficient for a limited analysis of nanospectra against small bacterial proteomes.

The software is publicly available at http://github.com/fenderglass/Nano-Align.

## Methods

### Signal acquisition from a sub-nanopore

**Fig 1:**
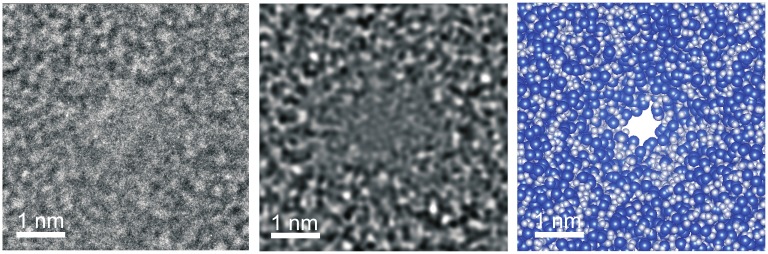
TEM micrograph of sub-nanopore is shown with a nominal diameter of 0.5 nm sputtered through silicon nitride membrane about 10-nm thick. The shot noise is associated with electron transmission through the pore. (center) Multi-slice simulations of the TEM image are consistent with the experimental imaging conditions. The simulations correspond to a bi-conical pore with a 0.5 x 0.4 *nm*^2^ cross-section and a 15 cone angle at defocus of -40 nm. (right) Space-filled model of the same pore is shown where the *Si* atoms are represented by spheres with a 0.235 nm diameter and *N* atoms by spheres with a 0.13 nm diameter. The scale bars are 1 nm.

The details regarding the experiments and methods used to acquire electrical current blockade signals from the translocation of single protein molecules through sub-nanopores have been described elsewhere [12]. To summarize, first, a pore with sub-nanometer cross-section was sputtered through thin silicon nitride membrane supported on a silicon chip using a tightly focused, high-energy electron beam in a scanning transmission electron microscope (Fig 1). The thickness of the membranes ranged from 8 to 12nm. Then the silicon chip supporting the membrane was embedded in a multiport microfluidic device that allowed for independent electrical access to the cis and trans-sides of the sub-nanopore by two *Ag/AgCl* electrodes. To perform electrical measurements, the sub-nanopore was immersed in 0.2 − 0.3 M *NaCl* and a transmembrane voltage in the range between 300 − 700 mV was applied. The resulting pore current was measured using an Axopatch 200B amplifier controlled with Clampex 10.2 software. Finally, recombinant denatured protein, along with 2 ⋅ 10 − 3% sodium dodecyl sulfate that imparted a nearly uniform negative charge to the protein, were added to the microfluidic reservoir (c.a. 20 fmoles of protein) and subsequently blockades in the open pore current associated with single molecules translocating through the pore were observed. It was determined that a lower transmembrane bias voltage improved the signal-to-noise ratio (SNR) and lengthened the median duration of the blockades, but it also increased the propensity for the pore to clog. Multi-level events associated with residual native protein structure or multiple molecules competing for the pore were occasionally observed, but were manually culled from the data pre- analysis [12].

Five proteins were analyzed by measuring the blockade currents through sub-nanopores: a recombinant chemokine CCL5 of length 68 AAs; two variants of the H3 histone designated as H3.2 and H3.3, which consist of the chain of 136 AAs, differing only by residue substitutions at positions 32, 88, 90 and 91; a tail peptide of the H3 histone (residues 1-20) and a fourth histone, H4 of length 103 AAs. More details about the datasets are given at the ‘Datasets’ section below.

### Signal pre-processing

**Fig 2:**
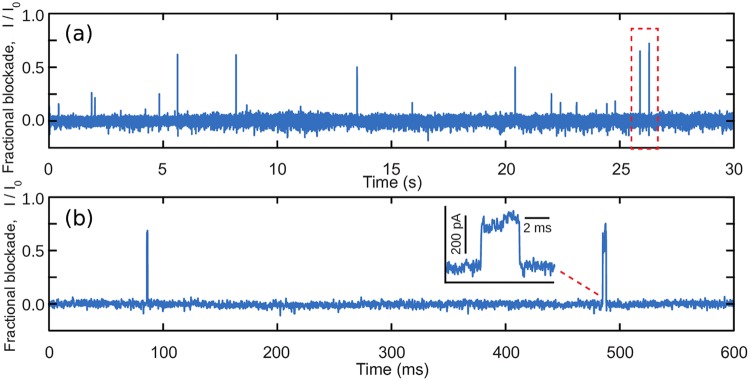
(a) An example of a pore current trace acquired from a denatured H3.3 histone translocating through sub-nanopore with a nominal diameter of 0.5-nm. (b) The bottom trace is a magnified view of a 600 ms region of a top trace, showing a current blockade associated with the translocation of a single protein molecule. In the figure, higher values correspond to larger blockade currents. Blockades, associated with the translocation of single proteins were identified as regions with fluctuations five standard deviations above the noise level and with duration > 1 ms.); and then the raw current *I* was converted into *fractional blockade current*.

When a single molecule of protein translocates through the sub-nanopore, its amino acids block the flow of ions, causing a change in the open pore current *I*_*open*_. The fraction of occupied pore volume *V*_*mol*_/*V*_*pore*_ (where *V*_*pore*_ and *V*_*mol*_ are volumes of the pore and molecule inside this pore, respectively) was assumed to be proportional to the *fractional blockade current*, which is calculated as |*I* − *I*_*open*_|/*I*_*open*_, where *I* is the raw current during the translocation. The raw signal measurements from the pore were pre-processed as follows: first, the discretized pore signal, sampled at 250 kHz, was split into the separate blockades, each one representing a translocation of a single protein (Fig 2)

Only events with sufficient duration to detect single-AA duration features were selected. Typical blockade duration analyzed here ranged from 1 to 20 milliseconds, as shorter times did not permit accurate discrimination of intra-event features due to the measurement bandwidth. The mean fractional blockade current varied from 0.05 to 0.5 for different nanospectra. Recorded signals exhibited fluctuations that were associated with different structural features of a protein translocating through the pore.

### Mean Volume model of protein translocation

Since the electrolytic current through the pore is associated with the occupied pore volume, one of the major factors that influences the signal is the volume of amino acids that occupy the sub-nanopore near the waist [19]. The estimates of amino acid volumes were obtained from crystallography data [20]. Since the pore can simultaneously accommodate multiple amino acids, it was assumed that the fluctuations in a blockade were proportional to a linear combination of amino acids volumes in the pore waist. In particular, we found that the mean volume of amino acids yielded a good approximation of the empirical signal values. Thus, given a protein *P* of length |*P*|, we split it into overlapping windows of size *k* (or *k*-mers) and generate a *theoretical nanospectrum* *MV*(*P*) as a vector of dimension |*P*| + *k* − 1 by taking the average volume of |*P*| − *k* + 1 *k*-mers and extra 2 * (*k* − 1) shorter prefix and suffix substrings from the beginning and end of a protein. These extra prefix and suffix substrings correspond to the start and the end of a translocation, when the pore is occupied by less than *k* amino acids. For example, for *k* = 3, the “protein” *KLMNP* results in a vector of length seven corresponding to the following substrings: *K, KL, KLM, LMN, MNP, NP*, and *P*.

Experimental analysis of peptides with post-translational modifications and mutations [12] revealed changes in the specific regions of the recorded signal traces, that corresponded to approximately four amino acids in length. In addition, simulations of the electric field in a 0.5x0.5 *nm*^2^ diameter, 8 *nm* thick pore in an *SiN* membrane indicated that the vast majority of the field was confined within 1.5 nm of the pore near the waist at the center of the membrane, which gives roughly the same estimate of the number of amino acids. Thus, the Mean Volume (MV) model assumes that each fluctuation in the blockade current corresponds to a read of a quadromer (short prefixes and suffixes of a protein correspond to shorter mers), which results in the best fit (among all reasonable values of *k*) with experimental nanospectra.

### Support Vector Regression-based model of protein translocation

**Fig 3:**
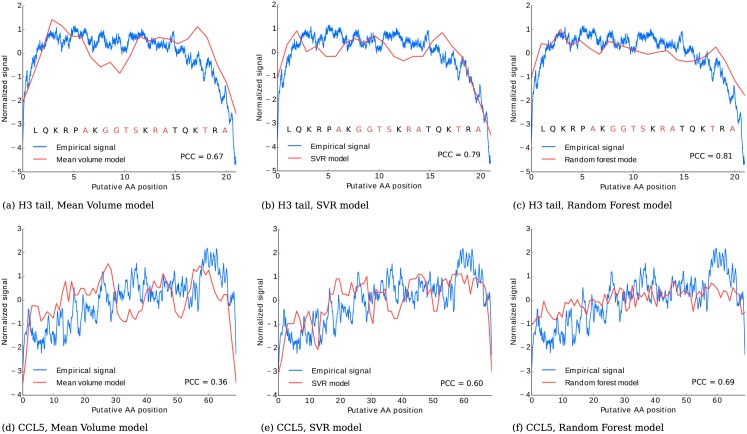
(a) A comparison of a consensus of 10 nanospectra of H3 tail peptide (10 AAs) and the corresponding theoretical nanospectra in the MV model (*k* = 4). As the coefficients of the linear dependence (for current vs. mean value) are unknown, each trace was normalized by subtracting the mean and dividing them by the corresponding standard deviation. Each signal position is associated with an amino acid under the assumption that protein translocation velocity is uniform. The poorly-matched regions are enriched with smaller amino acids (with volumes below the median, marked in red). The same comparison for the SVR model (b) and the RF model (c) shows better fit (measured as Pearson correlation coefficient). Similarly, comparison of a consensus of 10 nanospectra of CCL5 peptide (68 AAs) versus the MV, SVR, and RF models (d-f) shows improvement of the SVR and RF models over the MV model.

Generally, the MV model results in theoretical nanospectra correlated with the empirical data. The mean Pearson product-moment correlation coefficient between a consensus of experimental nanospectra (an average of multiple protein translocations, as described below) and the corresponding MV model was ranging from 0.25 to 0.45 for various datasets. However some regions show large deviations between theoretical and experimental nanospectra, which may be associated with additional attributes such as hydrophilicity or charge. In particular, our analysis revealed that such discordant regions were enriched with small amino acids, which have volumes below the median value (see Fig 3) for illustration and ‘Characterizing errors of the models’ section below for the detailed discussion). Since we acquired multiple nanospectra originating from multiple known proteins, an alternative approach for generating theoretical nanospectra was to use a supervised learning paradigm. We used a *Support Vector Regression* (an SVM-based regressor) to establish the correspondence between a *k*-mer inside the pore and a signal it generates [21]. Given an empirical nanospectrum *E* recorded from a protein *P*, we tiled *P* into overlapping quadromers *q*_*i*_ and discretized *E* into |*P*| + 3 points. Thus, each *q*_*i*_ had an associated experimental signal value *e*_*i*_.

**Fig 4:**
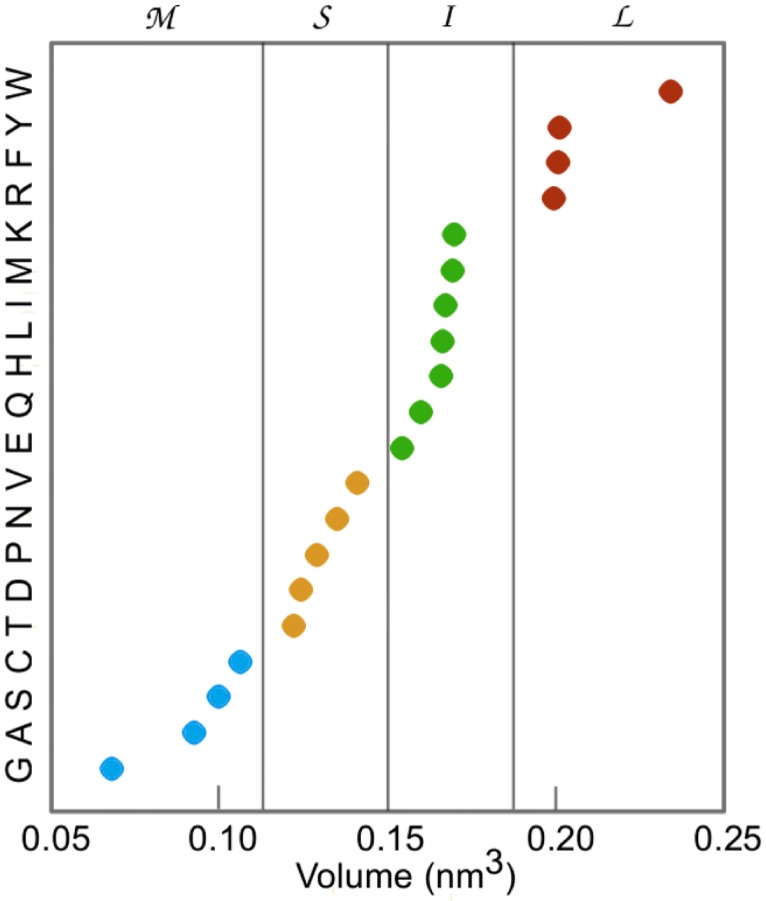
Amino acids separated into four categories based on their volume: G, A, S, C (Miniscule), T, D, P, N, V (Small), E, Q, H, L, I, M, K (Intermediate), and R, F, Y, W (Large).

Next, the feature space of the model has to be defined. Following the ideas of the MV model, it is natural to assume that blockade current is affected by the composition of amino acids in a quadromer, rather than their order (however, the dependence might be non-linear). As many of the 20 proteinogenic amino acids have similar volumes, we partitioned them into four volume groups (Fig 4) and defined a *feature vector* *f*_*i*_ of a quadromer *q*_*i*_ as the composition of amino acids from each group (as a tuple of length four). For example, an amino acid quardromer *GQLD* has zero amino acids from Large group (> 0.2*nm*^3^), two from Intermediate group (between 0.15 and 0.2 *nm*^3^), one from Small group (between 0.11 and 0.15*nm*^3^) and one from Minuscule group (< 0.11*nm*^3^), and is converted to a feature vector (0, 2, 1, 1). This choice of the feature space reduced the overfitting effect and increased coverage of the training dataset (there are only 35 distinct quadromer compositions in the defined feature space versus 20^4^ = 160 000 amino acid quadromers).

Using a set of pairs (*f*_*i*_, *e*_*i*_) we trained an SVR regressor with the Radial Basis Function kernel (implemented in an open-source library libsvm [22]). The *Support Vector Regression (SVR)* model takes a peptide *P* as input and outputs an SVR-based theoretical nanospectrum *SVR*(*P*) (Fig 3). The mean Pearson correlation coefficient between the theoretical and empirical nanospectra (consensus) for the SVRmodel was varying from 0.38 to 0.68 for different datasets, confirming the improvement over the MV model. The parameters of the SVR model were chosen through cross validation experiments and are equal to *C* = 1000, *γ* = 0.001, and *ϵ* = 0.01.

### Random Forest model of protein translocation

The analysis of error patterns of the SVR model revealed a bias in the signal estimation that was correlated with the hydrophilicity of the amino acids (see ‘Characterizing errors of the models’ section). Also, Bhattacharya et al. [23] recently reported that water molecules affect the signal of DNA translocating through the nanopore since hydrophilic amino acids are more likely to acquire a water molecule and change the effective volume [24]. Thus, it is desirable to include amino acid hydrophilicity into the model.

Motivated by these finding, we explored an alternative approach for supervised learning by using the *Random Forest (RF) regression* [25, 26] for theoretical nanospectra generation. In comparison to the SVR model, the resulting *Random Forest (RF)* model is more robust to outliers and exhibit less overfitting [27], which allowed us to use the volumes of all 20 amino acids as features. According to this RF model, each quadromer *q*_*i*_ from the training set is converted to a feature vector *f*_*i*_, where each element of the vector is a pair of volume and hydrophilicity of the corresponding amino acid.

We used an open source implementation of the Random Forest regressor from Scikit-learn package [28] to build the described model. The model performed well on the training sets, but the accuracy was poor on the test proteins with different amino acid sequence and composition. This was mainly caused by the fact that only a few among all possible amino acid quadromers were observed in the training sets. However, under assumption that nanopore current does not depend on the order of amino acids, it is possible to significantly expand the training sets by randomly permuting amino acids within quadromers. Specifically, prior to model we randomly permuted each *f*_*i*_ vector, leaving the same corresponding *q*_*i*_ value. This dataset expansion significantly improved the performance of the RF model on to training testing datasets. See Fig 3 for examples of theoretical nanospectra in the MV, SVR, and RF models.

### Protein identification

Given an experimental nanospectrum *S* and a protein *P*, we transformed *S* into a vector $\vec{S}$ by splitting *S* into |*P*| + 3 regions and taking the average value inside each of them. The vector $\vec{S}$ was then normalized by subtracting the mean and dividing by the standard deviation. Under the hypothesis that *P* has generated $\vec{S}$, we estimated the proportion of explained variance by computing *R*^2^ coefficient of determination between $\vec{S}$ and the model output. Given a database of proteins *DB*, a protein *P*(*S*, *DB*) is defined as a protein with the maximum *R*^2^ against *S* among all proteins from *DB*. A pair formed by the protein *P*(*S*, *DB*) and the nanospectrum *S* defines a putative *Protein-Nanospectrum Match (PrNM)*.

### Clustering nanospectra

**Fig 5:**
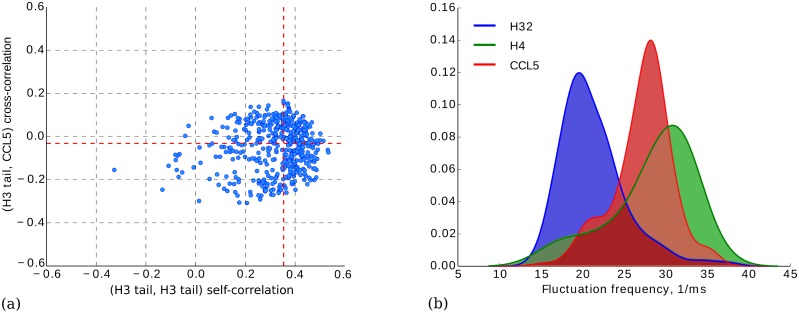
(a) Cross-correlations compared with self-correlations between the nanospectra originating from H3 tail protein and H4 protein. Cross-correlation values fluctuate around zero, while the median self-correlation is 0.35. (b) Distributions of fluctuation frequencies reveals peaks at positions 21 (H3.2), 34 (H4) and 29 (CCL5).

Single protein correlation analysis indicated that proteins were correlated more with themselves on average (Fig 5). In contrast, we did not observe such correlation in the open pore current, indicating that there is an inherent signal in blockades. However, electrolytic current through the pore is affected by many factors, such as uncorrelated time-dependent fluctuations in the ionic current and electrical instrument noise, which results in noisy nanospectra. Averaging multiple nanospectra from the same protein resulted in significant noise reduction and increased accuracy of PrNM identification. This effect is similar to improvements in peptide identifications that are achieved by clustering of mass spectra in traditional proteomics [29, 30].

Typically, clustering of 5 − 10 nanospectra results in a consensus nanospectrum that significantly improves the signal-to-noise ratio over a single nanospectrum (the mean Pearson correlation coefficients between theoretical and empirical nanospectra increased 1.5—2-fold for various datasets). Since each of the existing datasets of nanospectra originated from a single pure protein, we randomly partitioned the dataset of nanospectra into clusters and performed identification of consensus nanospectra instead of a single nanospectrum.

### Estimating protein length based on a blockade signal

In traditional proteomics, the precursor mass assists top-down protein identification since it greatly reduces the computational space that has to be searched in the protein database. Likewise, information about the protein length would be very useful for SMPI, but estimating the protein length based on a nanospectrum originating from a sub-nanopore is a non-trivial problem since the existing experimental protocol does not control the translocation speed that may vary widely as evident from the blockade duration.

Our analysis revealed that protein translocations modulate the blockade current, which was captured by the measurements. Each blockade, associated with the translocation of a protein showed a characteristic number of fluctuations during the duration of the blockade. It turned out that the fluctuation frequency (described below) was correlated with the protein length and the other features, such as amino acid composition.

We explored a possibility of the separation of a sample of nanospectra into clusters corresponding to different proteins. From a sample of different proteins, we estimated the *fluctuation frequency* of each nanospectrum as the number of peaks (local maximums) divided by the duration of the blockade. The distribution of fluctuation frequencies (Fig 5b) revealed that each protein in our datasets has a characteristic peak in the distribution. To separate the nanospectra into clusters based on the fluctuation frequency one can apply the Gaussian Mixture model to estimate the protein lengths from nanospectra and to improve the efficiency of SMPI.

### The challenge of analyzing protein mixtures

Analyzing a mixture of multiple proteins is conceptually harder than analyzing the existing experimental datasets of nanospectra that all originated from pure protein solutions. Since it is unknown what protein gives rise to what nanospectrum in a mixture, it is difficult to cluster nanospectra for a reliable identification. Further, orientation of each molecule must be deduced prior to clustering since each protein can translocate through the pore in two different directions.

However, it is possible to cluster nanospectra based on their estimated fluctuation frequency to differentiate proteins with different lengths. As multiple proteins may have a similar length, it is important to further split some *length-based clusters* into finer *protein-based clusters*. We believe, that this could be done by applying clustering algorithms which automatically estimate the number of clusters (e.g. Affinity Propagation [31]). Evaluating the results of clustering in the case of complex mixtures was problematic since all available experimental datasets of nanospectra were generated from the pure protein solutions.

## Results

### Datasets

We benchmarked Nano-Align using nanospectra from five short human proteins: H3.2, H3.3, H4, CCL5 and H3 tail peptide (Table 1). The nanospectra from H3.2, H3.3 and H4 were acquired using the two similar pores whereas the nanospectra for CCL5 and H3 tail were acquired using two different pores with different sizes. The proteins were split into three pairs: (CCL5, H3 tail), (H4, H3.2) and (H3.3, H3.2). For each pair of proteins, the SVR and RF models were trained using the protein with higher number of nanospectra and the accuracy of identifications was estimated using the other protein from the pair. The first two pairs represented proteins that were very different in both length and amino acid composition, thus minimizing the overfitting effect. The third pair represented highly similar proteins, that only differ in four amino acids.

**Table 1:** Datasets summary.

Dataset	Peptide length	#Nanospectra	Pore id	Pore size (nm^2^)
H3.2	136	445	ZD349, ZD350	0.6x0.5
H3.3	136	25	ZD349	0.6x0.5
H4	103	89	ZD350	0.6x0.5
CCL5	68	239	ZD158	0.8x0.6
H3 tail	20	477	ZD220	0.6x0.5

### Evaluating protein identification accuracy

To evaluate the accuracy of SMPI, we constructed decoy protein database for each dataset from the correct protein and randomly generated proteins of the same length and amino acid composition as the correct protein. The size of decoy database varied from 10^5^ to 5 ⋅ 10^6^ for different datasets, depending on the identification accuracy and the number of nanospectra in the dataset. The p-value of a PrNM was approximated as the percentage of proteins from the database scoring higher than the correct protein against the given nanospectrum.

**Fig 6:**
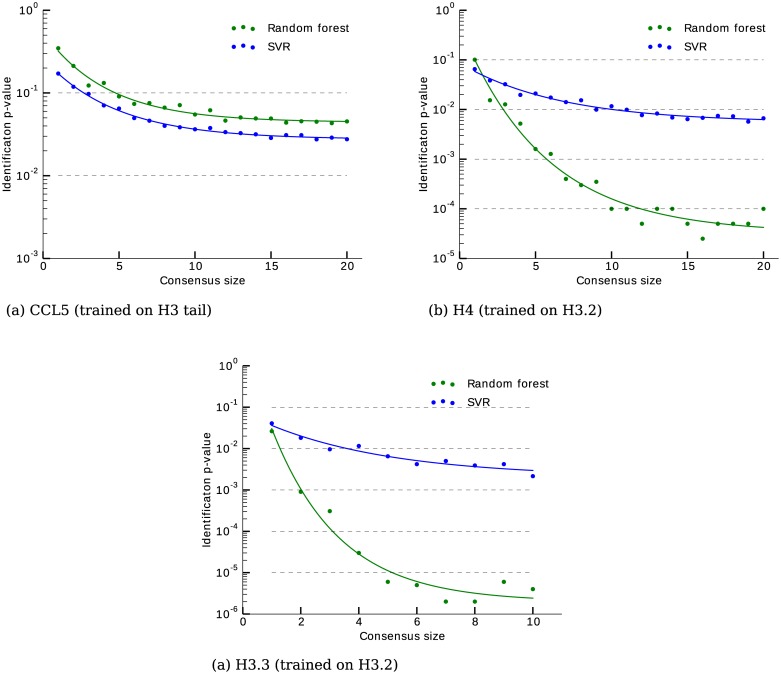
(a) Cross-correlations compared with self-correlations between the nanospectra originating from H3 tail protein and H4 protein. Cross-correlation values fluctuate around zero, while the median self-correlation is 0.35. (b) Distributions of fluctuation frequencies reveals peaks at positions 21 (H3.2), 34 (H4) and 29 (CCL5).

Below we show results for the SVR and RF models only since they turned out to be significantly more accurate than the MV model for all datasets. Fig 6 shows median p-values for SVR and RF models as a function of the number of nanospectra in a cluster. As expected, both models showed the improvement in the accuracy with the increase in the cluster size. The p-values for the pair (CCL5, H3 tail) were high for both models (0.03–0.05 for a consensus of size 10). However, the dataset (H4, H3.2) showed a significant improvement for the RF model (p-values of the order of 10^−4^ for a consensus of 10 nanospectra), while the accuracy of the SVR model was comparable to the previous dataset. Finally, the RF model showed high accuracy on (H3.3, H3.2) dataset, with p-values below 10^−5^ for the nanospectra clusters of size five.

The RF model consistently outperformed the SVR model on the datasets that were generated using pores of similar sizes, which suggests that the decision trees are better suited for SMPI due to their robustness against outliers. Also, amino acid hydrophilicity proved to be a valuable predictor of the pore signal. The RF model performed slightly worse than the SVR model on the dataset generated using two different pores, suggesting that it is more sensitive to the experimental conditions. The fact that the RF model performed better on the proteins that were more similar to the training proteins is not surprising, but rather highlights the importance of choice of the training set, which should have substantial coverage of the data.

Additionally, we benchmarked the RF model performance using a database containing real human proteins. We extracted all proteins of length between 100 and 160 from the human proteome (about 20% of the human proteome) and performed the identification of H3.3 spectra against this reduced database. On average, the true protein was ranked five against all other proteins (for a cluster of size five). An example of database hits is given in the Table 2. Interestingly, all high-scoring proteins belong to H3 histone family and differ by only few amino acids. While the search space was artificially reduced, this experiment already provides a justification for analysis of unknown nanospectra against small bacterial proteomes, after further improvements in the protein length estimation discussed above.

**Table 2:** An example of H3.3 nanospectra identification (for a cluster of size five) against all human proteins of length 100–160 AAs. The total database size is 14 293, which covers approximately 20% of the human proteome taken from the UniProt database. The correct protein (H3F3A) is shown in bold. Proteins from the H3 family exhibit the highest *R*^2^ scores among other proteins from the database.

Rank	Protein Id	*R*^2^ score	Length
1	H3F3B	0.4002	132
2	HIST2H3A	0.3989	136
3	HIST1H3A	0.3980	136
4	H3F3C	0.3905	135
**5**	**H3F3A**	**0.3871**	**136**
6	HIST3H3	0.3819	136
7	HIST2H3PS2	0.3714	136
8	PRR14	0.3248	104
9	BRD8	0.3146	122
10	ANAPC16	0.3028	110

### Characterizing errors of the models

**Fig 7:**
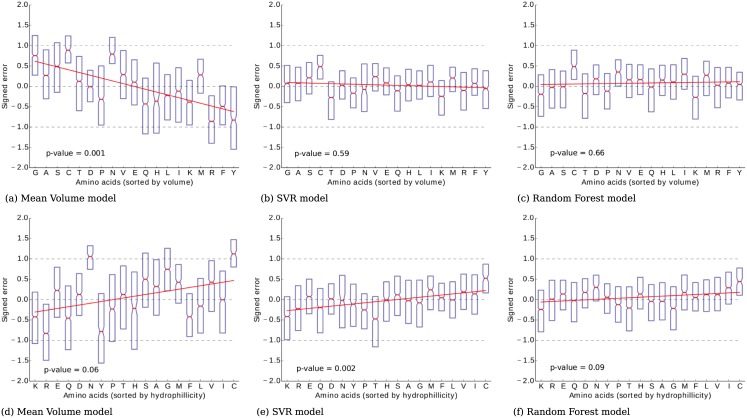
Error (calculated as the difference between empirical and theoretical nanospectra) for amino acids of H3.2 protein sorted in the increasing order of their volumes. (a) MV model has a tendency to underestimate signal associated with small amino acids and overestimate signal from large amino acids. SVR model (b) and RF model (c) trained on H3.2 dataset do not have volume-related bias. As *Trp* is not present in H3.2, it is not shown on the figure. P-values are given for the hypothesis that linear slope is non-zero. Similar analysis reveal signal bias with respect to amino acid hydrophilicity for SVR model (e). MV model (d) and RF model (f) do not show statistically significant bias.

For each of the three models (MV, SVR and RF) we measured the bias with respect to different features of amino acids. Using H3.2 dataset (that provides the best amino acid coverage) we calculated the *signed error* defined as the mean difference between the empirical and theoretical nanospectra. For each amino acid, the signed error was measured among the associated quadromers. Fig 7 shows the volume-related bias of the MV model. This bias could be explained by the fact that larger amino acids have more influence on the pore signal than smaller amino acids. The SVR model and RF model show no bias with respect to amino acid volumes. A similar analysis revealed a bias with respect to amino acid hydrophilicity in the SVR model. The MV model did not show a clear dependence, possibly due to the dominant effect of the volume bias. The RF model showed no statistically significant bias related to hydrophilicity.

## Discussion

We presented the first algorithm for Single Molecule Protein Identification using a signal generated by a protein translocation through a sub-nanopore. We also proposed three models for generating theoretical nanospectra and concluded that the Random Forest model results in the most accurate identifications. The typical estimated p-values of identification accuracy were ranging from 10^−4^ to 10^−6^, which is already sufficient for a limited analysis of nanospectra against small bacterial proteomes containing a few thousands proteins. The comparison of algorithm performance on different datasets suggests that the model sensitivity will further improve when more nanospectra originated from different proteins become available.

Cysteine (Cys) was the highest source of error in all three models for H3.2. Likewise, Cys was an above average source of error in CCL5 [12] but, it was a below average source of error in the similar sequence of CXCL1. Thus, it seemed unlikely that only the size affects the error. On the other hand, both Cys and Met, which exhibit higher number of prediction errors are at the high end of the hydropathy index and have only few waters (4 and 10, respectively) binding them [32], which may indicate that water affects the blockade current. In addition, it has been speculated that charge could also affect the duration and magnitude of a blockade [12, 33]. Whereas it seems likely that both charge and water play a role in the blockade current, measurements and the MV model testing these ideas have been inconclusive so far.

While SMPI is currently not in a position to compete with top-down proteomics, this technology is still in its infancy. Furthermore, due to the inherent single molecule sensitivity, there are several avenues of research that can be addressed uniquely by SMPI that offer protein-discrimination from very small samples (attomoles). Thus, SMPI has a potential to emerge as a new technology for accurate protein identification.
